# Myostatin Mutation Promotes Glycolysis by Increasing Phosphorylation of Phosphofructokinase *via* Activation of PDE5A-cGMP-PKG in Cattle Heart

**DOI:** 10.3389/fcell.2021.774185

**Published:** 2022-01-28

**Authors:** Mingjuan Gu, Xinyu Zhou, Lin Zhu, Yajie Gao, Li Gao, Chunling Bai, Lei Yang, Guangpeng Li

**Affiliations:** ^1^ State Key Laboratory of Reproductive Regulation and Breeding of Grassland Livestock, Inner Mongolia University, Hohhot, China; ^2^ School of Life Science, Inner Mongolia University, Hohhot, China; ^3^ Baotou Teachers’ College, Baotou, China

**Keywords:** heart, glycolysis, PDE5A-cGMP-PKG, phosphofructokinase, myostatin mutation

## Abstract

Myostatin (MSTN) is a primary negative regulator of skeletal muscle mass and causes multiple metabolic changes. However, whether MSTN mutation affects heart morphology and physiology remains unclear. Myostatin mutation (MT) had no effect on cattle cardiac muscle in histological examination, but in biochemical assays, glycolysis increased in cattle hearts with MT. Compared with wild-type cattle, there were no differences in mRNA and protein levels of rate-limiting enzymes, but phosphofructokinase (PFK) phosphorylation increased in cattle hearts with MT. Transcriptome analysis showed that phosphodiesterase-5A (PDE5A), a target for inhibiting cGMP-PKG signaling, was downregulated. For the mechanism, chromatin immunoprecipitation qPCR showed that the SMAD2/SMAD3 complex in the canonical downstream pathway for MSTN combined with the promoter of PDE5A. The cGMP-PKG pathway was activated, and PKG increased phosphorylation of PFK in cattle hearts with MT. In addition, activation of PKG and the increase in PFK phosphorylation promoted glycolysis. Knockdown of PKG resulted in the opposite phenomena. The results indicated that MT potentiated PFK phosphorylation *via* the PDE5A-cGMP-PKG pathway and thereby promoted glycolysis in the heart.

## Introduction

Myostatin (MSTN), also called growth differentiation factor 8, is a member of the transforming growth factor beta superfamily (Matsakas and Diel, 2005). It is primarily expressed in skeletal muscles and negatively regulates muscle development and growth (Matsakas and Diel, 2005). The importance of MSTN is most dramatically demonstrated by the overt muscle hypertrophy that results from inactivation of MSTN by engineered deletion or natural mutation in human, cattle, and mice (McPherron and Lee, 1997; Rodgers and Garikipati, 2008). Myostatin is translated as a precursor protein, and the active, mature MSTN peptide is finally produced *via* three proteolytic processing events (Cotton et al., 2018). Mature MSTN dimers combine with activin receptor IIB (ActRIIB) and then in turn phosphorylate type I receptors, including activin receptor-like kinase 4 (ALK4) or activin receptor-like kinase 5 (ALK5) and Smad 2 and 3 transcription factors (McPherron et al., 1997; Rebbapragada et al., 2003; Han et al., 2013). Activated Smad2/3 recruits Smad4 in the cytoplasm, and the complex translocates into the nucleus and modifies the transcription of the genes involved in cellular differentiation and proliferation (Langley et al., 2002; Dong et al., 2017; Gao et al., 2020). In addition to expression in skeletal muscle, MSTN is produced in the heart (Sharma et al., 1999), and in the heart of animals with an MSTN mutation (MT), MSTN expression decreases significantly (Luo et al., 2019). Hence, mutation of the MSTN gene most likely has an important role in the heart, in addition to playing a major role in the regulation of muscle metabolism and growth (Shyu et al., 2006). However, the mechanism involved in the effect of MT on the heart has not been fully investigated.

Glycolysis is universal in cells of an organism, and the enzymes needed for glycolysis are in the cytosome. Glycolysis can occur in the absence of oxygen (anaerobic) or in the presence of oxygen (aerobic), but products are different. Lactic acid is the final product in anaerobic conditions, whereas in the aerobic conditions, pyruvate is formed and eventually oxidized to CO_2_ and H_2_O (Bacci et al., 1985). Myostatin also plays a crucial role in regulating glycolytic processes (Chen et al., 2010). In pig, deficiency of MSTN results in an increase in the amount of fast glycolytic fibers (Xing et al., 2017). Xin et al. (2020) reported that when MSTN is knocked out in cattle, the activity of many key enzymes involved in glycolytic processes to regulate glucose increases (Xin et al., 2020). In addition, cardiomyocyte-specific MSTN deletion in the postnatal mice hearts increases glycolytic capacity (Biesemann et al., 2014). To date, however, the consequences of MT for glycolysis in hearts of larger animals have not been examined.

Phosphodiesterase-5A (PDE5A) is one of 11 isoforms in the phosphodiesterase superfamily. It is cardiomyocyte-specific and hydrolyzes 3′,5′-cyclic guanosine monophosphate (cGMP) (Kim and Kass, 2017), which is a critical secondary messenger molecule. The cGMP drives activation of protein kinase cGMP-dependent (PKG), which regulates diverse biological processes by phosphorylation of target substrates and has an important role in protecting the heart (Lee and Kass, 2012). Activation of cGMP-PKG signaling has been used in therapeutic strategies for heart failure (Nakamura and Tsujita, 2021). Moreover, PKG prevents/blocks adverse cardiac reactions activated by rapamycin complex-1 under pathological growth conditions by phosphorylating tuberin (Ranek et al., 2019). However, the role of cGMP-PKG in hearts with MT remains unclear.

In this study, we examined cardiac changes in cattle with MT that we had previously generated with CRISPR/Cas9 technology. The PDE5A-cGMP-PKG pathway was activated in cattle hearts with MT. It was hypothesized that there was most likely a link between MT and glycolysis *via* the PDE5A-cGMP-PKG signaling pathway. The results indicated that MT promoted glycolysis in cattle hearts by increasing phosphorylation of phosphofructokinase (PFK) by regulating PDE5A-cGMP-PKG with attenuation of SMAD2/SMAD3.

## Materials and Methods

### Animals

As in our previous report (Gao et al., 2020), we used CRISPR/Cas9 and somatic cell nuclear transfer to generate MSTN knockout cattle. In the study, the cattle with MT used were a cross between wild-type female Luxi cattle and male MSTN^−/−^ cattle. A total of 30 cattle, 18 MSTN mutant cattle (9 female and 9 male), and 12 wild-type cattle (6 female and 6 male) were used. The cardiac samples were derived from 24-month cattle.

### Histological Staining

After the animals were sacrificed, the hearts were rapidly harvested and the samples were fixed with 4% paraformaldehyde and embedded in paraffin. Sections (5 µm thick) were taken for hematoxylin and eosin (H&E) staining at each time. Sections were also deparaffinized and hydrated in a series of graded ethanol for further study, including staining with Sirius Red to assess collagen deposition to depict fibrosis. Finally, a quantitative digital image analysis system (Image-Pro Plus 6.0) was used for quantitative analyses.

### Immunofluorescence

Paraffin-embedded heart sections were fixed with 4% paraformaldehyde in PBS (pH 7.4), permeabilized by incubating in PBS containing 0.3% Triton X-100, and blocked with 5% bovine serum albumin (BSA). After that, paraffin-embedded heart sections were incubated with antibody MSTN (Abcam, United States, ab124721) overnight at 4°C, and then incubated with the appropriate secondary antibody (Abcam, United States, ab150081) for 30 min at 37°C. All sections were counterstained with DAPI (Thermo, United States). Specific fluorescence was imaged by laser-scanning confocal microscopy (Carl Zeiss, Germany, LSM710).

### Cell Culture and Treatment

H9C2 cardiomyocytes were purchased from the Cell Bank of Chinese Academy of Science (Beijing, China). Cells were cultured in Dulbecco’s modified Eagle’s medium (DMEM; Gibco, United States) containing 10% inactivated fetal bovine serum (FBS; Gibco, United States) at 37°C in a humidified incubator with 5% CO_2_. PKG short hairpin RNA (shRNA) was purchased from Sangon Biotech (China). SMAD3 shRNA and GDF-8 shRNA were purchased from Santa Cruz (sc-383774-SH and sc-39775-SH). PKG activator 8-Br-cGMP was purchased from Abcam (ab141449). Lipofectamine LTX Reagent (Thermo, United States) was utilized for transfection. After transfection for 48 h, the efficiency of transfection was monitored by Real-time PCR and Western blotting, and 100 μM 8-Br-cGMP was added to the upper compartment.

### Western Blot

The total protein was extracted from the left ventricular myocardium of the cattle and lysed in pre-cooled Radio Immunoprecipitation Assay (RIPA) buffer with protease inhibitors. The tissue lysate was centrifuged at 8,000 × *g* for 30 min at 4°C, and the supernatant was taken for Western blotting. Proteins were separated on 10% SDS-polyacrylamide gel and transferred to NC membranes by electroblotting. The membranes were blocked with 5% non-fat milk in Tris-buffered saline with 0.1% Tween-20 (TBST) at room temperature for 1 h and incubated with anti-MSTN (Abcam, United States, ab201954), anti-SMAD2 (Abcam, United States, ab33875), anti-SMAD3 (Abcam, United States, ab40854), anti-SMAD2+3 (Abcam, United States, ab202445), anti-SMAD4 (Abcam, United States, ab40759), anti-TGF beta Receptor I (Abcam, United States, ab31013), anti-ACVR2B (Abcam, United States, ab76940), anti-PFK (Proteintech, United States, 55028-1-AP), anti-HK2 (Proteintech, United States, 22029-1-AP), anti-PK (Proteintech, United States, 15821-1-AP), anti-PDE5A (Proteintech, United States 22624-1-AP), anti-PKG (Proteintech, United States, 21646-1-AP), and anti-phosphoserine (Abcam, United States, ab9332) in TBST containing 0.5% non-fat milk at 4°C overnight. According to the resistance of primary antibody, horseradish peroxidase-conjugated goat anti-mouse and anti-rabbit secondary antibodies (1:10,000) were used to incubate the membranes at room temperature for 1 h, respectively, followed by detection using the chemiluminescence labeling detection reagent ECL Plus (Thermo, United States, 32209). Quantification analysis of blots was performed with the ImageJ (1.8.0) software.

### ELISA Assay

The clear supernatant extract of heart tissues was collected and used for ELISA using cGMP kits following the manufacturer’s instructions (Abcam, United States, ab133052). Samples were analyzed in triplicate.

### Real-Time PCR

Total RNA from the H9C2 cells and heart tissue were isolated using the RNAiso Plus kit (Takara, Japan, 9108). cDNA was synthesized by a PrimeScript RT reagent Kit with gDNA Eraser (Perfect Real Time) (Takara, Japan, RR047A). We used ABI7500 real-time PCR (Applied Biosystems, United States) and SYBR Green (Takara, Japan, RR820A) to amplify the cDNA. Primer sequences were as tabulated in Table S1. The PCR amplification protocol was as follows: 95°C for 30 s, followed by 40 cycles at 95°C for 5 s, and 60°C for 34 s. For normalization, housekeeping gene *GAPDH* was used as the internal control and fold changes in gene expression were determined using the comparative threshold cycle (2^−∆∆Ct^) method.

### Biochemical Detection

Enzyme activities and metabolites were assayed using hexokinase (HK) activity assay kit, PFK activity assay kit, pyruvate kinase activity assay kit, FDP Fructose-1,6 diphosphate (FDP) kit, and Glucose-6-phosphate (G6P) kit according to the manufacturer’s protocols from Comin (China). The optical densities were measured using a microplate reader (Thermo, United States).

### Transcriptomic Analysis

The mapped fragments were standardized using the fragments per kilobase per million reads (FPKM) method. DEG between MT and WT cattle was identified by the DEG-seq software package applying the MA-plot-based method with random sampling (MARS) model methods. The *p*-value < 0.05 and the absolute value of fold change >2 were considered to have significant expression abundance. All DEGs were mapped to terms in KEGG database. The raw sequence data reported in this paper have been deposited in the Genome Sequence Archive in BIG Data Center, Beijing Institute of Genomics (BIG), Chinese Academy of Sciences, with the accession number CRA004316 that are publicly accessible at https://bigd.big.ac.cn/gsa/browse/CRA004316.

### Chromatin Immunoprecipitation Assay

Chromatin immunoprecipitation (ChIP) was operated according to the guidelines by the SimpleChIP Plus Enzymatic Chromatin IP Kit (Magnetic Beads) (Cell Signaling Technology, United States, 9005). Chromatin was crosslinked and immunoprecipitated with 2 μg of anti-SMAD2+SMAD3 (Abcam, United States, ab202445) and 30 μl of protein G beads at 4°C overnight. The negative control was normal rabbit IgG. Lastly, the purified immunoprecipitated chromatin was analyzed by quantitative real-time PCR. The sequences of the primers are provided in Supplementary Table S1.

### Co-Immunoprecipitation

To investigate the phosphorylation level of the key enzyme protein in MT heart, we detected the phosphorylated HK2, PFK, and PK protein in MT and WT heart. Since no commercial phosphorylation antibodies of HK2, PFK, and PK protein were available, the pan phosphorylation antibody was used to study the phosphorylation status of key enzymes in MT and WT cattle heart by immunoprecipitation (IP) analysis. Briefly, HK2, PFK, and PK were pulled down with the anti-HK2, PFK, and PK antibody, and an IP/Western blot assay was carried out to analyze the phosphorylation of HK2, PFK, and PK, as a previously reported method (Lin et al., 2012; Deshpande et al., 2021). Specific methods of co-IP were described as follows. Lysates of cattle heart tissue and H9C2 cells were respectively extracted with proteinase inhibit cocktail Complete Mini (Thermo, United States) and phosphatase inhibitor cocktail PhosSTOP (Thermo, United States). The total protein concentrations were quantified by the Pierce BCA Protein Assay Kit (Thermo, United States). Co-immunoprecipitation (co-IP) was performed using the Thermo Scientific Pierce co-IP kit (#26149). Ten micrograms of the antibody (Anti-PFK, Anti-HK2, Anti-PK, and Anti-PKG) were incubated with the delivered resin and covalently coupled. The antibody-conjugated resin was incubated with 200 µl of the whole cattle heart tissue and H9C2 cells protein lysates overnight at 4°C, respectively. The resin was washed and the protein complexes bound to the antibody were eluted. Subsequent Western blot analyses were performed as described previously.

### Luciferase Reporter Assay

The promoter region of the PDE5A gene was amplified from the bovine genomic DNA and inserted into the pGL3-Basic vector (Promega, United States). HEK293T cells were seeded and transfected. Forty-eight hours later, luciferase activity was assessed by a Dual luciferase Reporter Assay System (Promega, United States).

### Statistical Analysis

Comparisons between two observations in the same subjects were assessed by Student’s paired *t*-test. Results were expressed as the mean ± standard deviation (SD). *p*-value of less than 0.05 was accepted as statistical significance.

## Results

### Anatomic Morphology of the Heart in Wild-Type Cattle and Those With Myostatin Mutation

**FIGURE 1:**
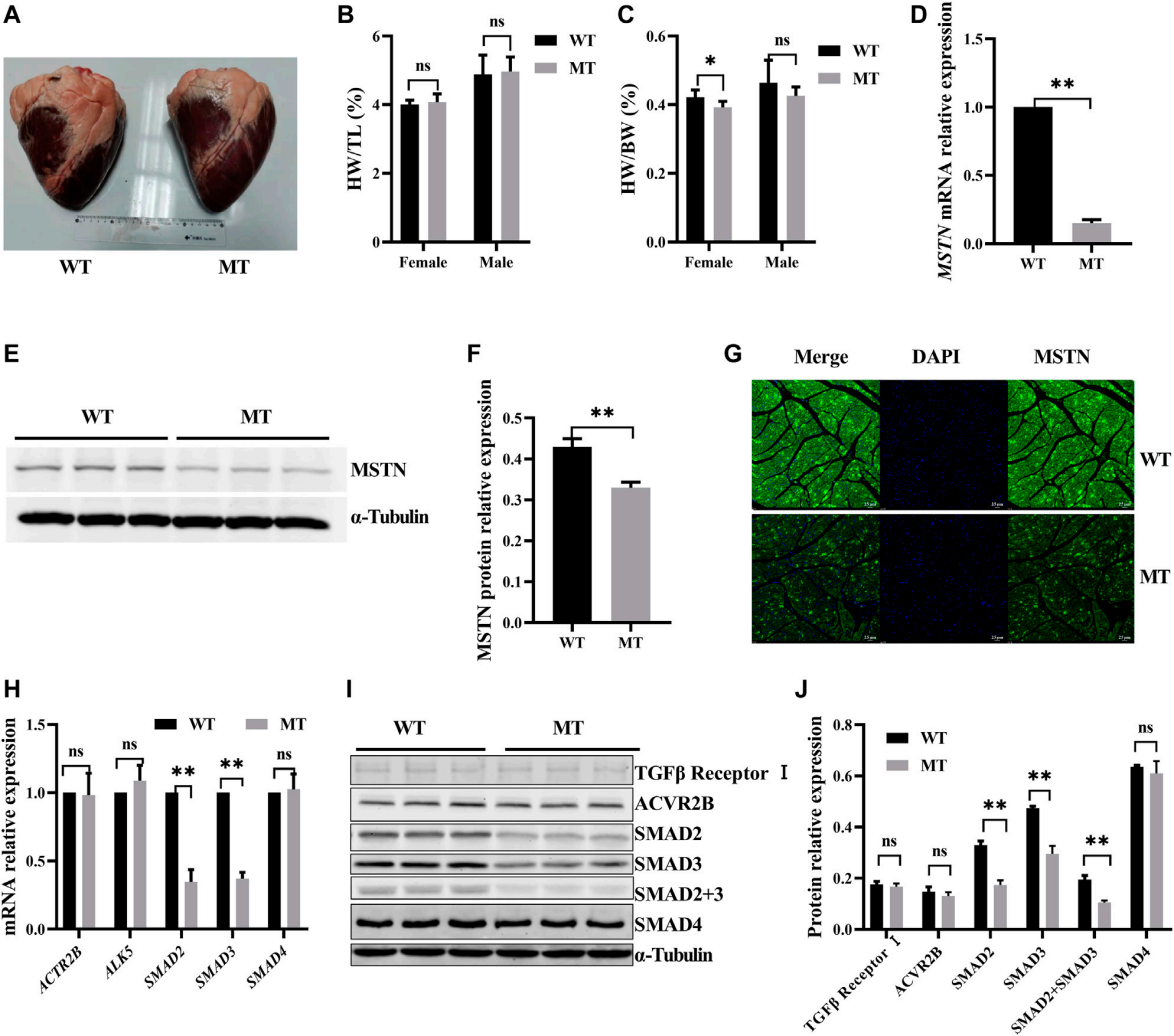
Myostatin mutation decreased SMAD2/3 expression but had no effect on cardiac anatomy of cattle. **(A)** Whole heart images of MT and WT cattle. **(B,C)** Statistical results of heart weight (HW)/tibial length (TL) and HW/body weight (BW) in MT and WT cattle. **(D)** Expression of MSTN mRNA in heart of MT and WT cattle. **(E,F)** Expression of MSTN protein in heart of MT and WT cattle. **(G)** Representative expression of MSTN immunofluorescence images of heart (green, MSTN; blue, nuclei; scale bar, 25 μm). **(H)** Expression of MSTN signaling pathway factor mRNA in heart of MT and WT cattle. **(I, J)** Expression of MSTN signaling pathway factor protein in heart of MT and WT cattle. All data are presented as mean ± SD. Compared with the WT group, *p < 0.05, **p < 0.01, ns: no significant; *t*-tests were used to calculate the *p*-values.

To determine the effect of MT on cardiac morphology, the anatomic structure of the heart was assessed. Thirty hearts were analyzed, including12 from wild-type (WT) cattle and 18 from those with MT. As shown in Figure 1A, no obvious exterior morphological abnormalities were observed on hearts in either group. Weight, volume, left ventricular wall thickness, right ventricular wall thickness, left atrium wall thickness, right atrium wall thickness, interventricular septum wall thickness, and interatrial septum wall thickness of hearts with MT cattle without normalization were heavier or thicker than the those of same sex WT hearts (Supplementary Table S2). Female and male cattle with MT had 3.5% and 5% increases in mean heart weights (HW) compared to WT cattle. There was no statistical difference after normalization with tibial length (TL), which indicated that cattle with MT had relatively larger hearts than those of WT cattle (Figure 1B). Conversely, skeletal muscle with MT cattle weight normalized to tibia length was significantly higher than those of WT cattle (Supplementary Figures 1A,B). The ratio of heart weight (HW) to body weight (BW) (HW/BW) of the female WT cattle was higher than those of cattle with MT (Figure 1C), possibly because the body weight of cattle with MT was significantly higher than that of WT cattle (Supplementary Table S2).

### Myostatin Mutation Decreased SMAD2/3 Expression

Effects of MT on expression of cardiac MSTN signaling pathway factors were evaluated. Compared with the WT cattle, expressions of MSTN mRNA and protein were decreased significantly in cattle hearts with MT (Figures 1D–F). Immunofluorescence staining also showed that MSTN signals decreased significantly in cattle hearts with MT (Figure 1G). The results indicated that expression of MSTN decreased significantly in cattle hearts with MT. However, expression of both mRNA and protein of the ActRIIB/ALK5 heterodimer, the predominant receptor of MSTN, was not significantly different between WT hearts and those with MT (Figures 1H–J). Moreover, we examined the expression of SMAD2, SMAD3, and SMAD4 in the canonical pathway for MSTN and found that SMAD2 and SMAD3 were significantly lower in cattle hearts with MT than in WT hearts, whereas SMAD4 had no difference (Figures 1H–J). The results suggested that MT decreased the expressions of MSTN, SMAD2, and SMAD3.

### Effects of Myostatin Mutation on Cardiac Histological Structures

**FIGURE 2:**
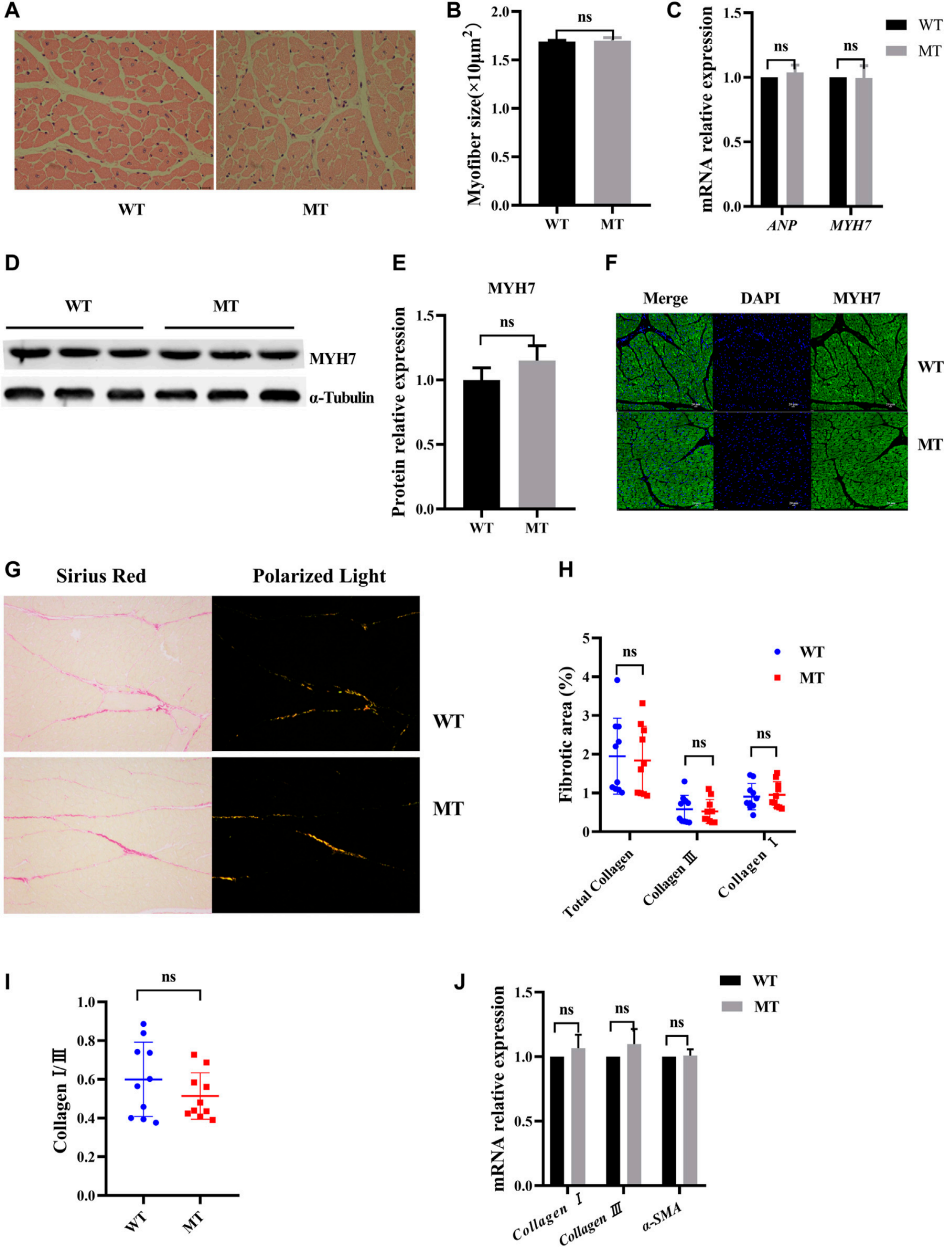
Myostatin mutation did not cause cardiac histology change. **(A)** Hematoxylin and eosin (HE) staining of heart tissue samples (400×). **(B)** Calculated cross-sectional area of heart samples (*n* > 100 cells for each group). **(C)** mRNA levels of the hypertrophic markers. **(D,E)** Expression of MYH7 protein in heart of MT and WT cattle. **(F)** Representative expression of MYH7 immunofluorescence images of heart (green, MYH7; blue, nuclei; scale bar, 25 μm). **(G)** Representative images of Sirius Red-stained sections and polarized light microscopy from MT and WT cattle. **(H,I)** Analysis of cardiac fibrosis, and in polarized light collagen type I (red-yellow) and collagen type III (green) in MT and WT cattle. **(J)** mRNA levels of the fibrosis markers. All data are presented as mean ± SD. Compared with the WT group, *p < 0.05, **p < 0.01, ns: no significant; *t*-tests were used to calculate the *p*-values.

To assess the effect of MT on cardiac histology, the cardiomyocyte cross sections and the composition of cardiac collagen were examined. In HE staining, no significant difference in cardiomyocyte surface area was identified between WT cattle and those with MT (Figures 2A,B, and Supplementary Figure S1C). Expressions of the cardiac hormone gene *ANP* and the cardiac structural gene *MYH7* were not significantly different in the cardiac tissues between WT cattle and those with MT (Figure 2C). Protein levels of MYH7 were also not different (Figures 2D,E). Immunofluorescent staining for MYH7 also showed no significant differences (Figure 2F). The composition of cardiac collagen was determined by Sirius Red staining and observation under a polarized-light microscope. The collagen area of heart tissues in cattle with MT was not significantly different compared with that in WT cattle (Figures 2G,H and Supplementary Figure S1D). As expected, in cattle with MT, there were no changes in collagen types I and III or the ratio of type I to III, compared with WT cattle (Figures 2H,I). In addition, reverse-transcription qPCR confirmed that expression of *collagen I*, *collagen III,* and *α-SMA* did not increase significantly in heart tissues of cattle with MT (Figure 2J). These data suggested that MT did not cause changes in cardiac histology.

### Myostatin Mutation Promoted Glycolysis in the Heart

**FIGURE 3:**
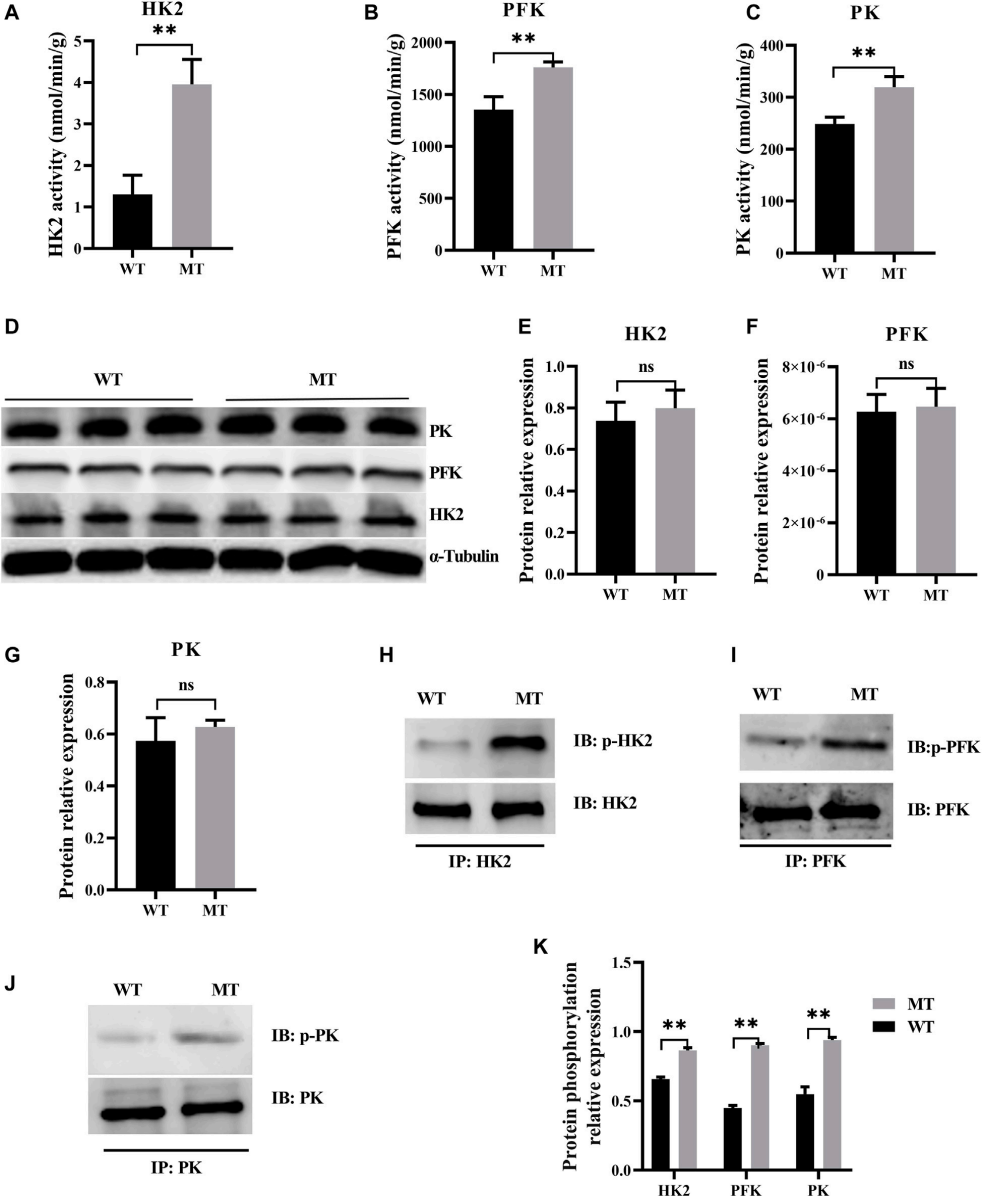
Myostatin mutation mutant effect on heart glycolysis of cattle. **(A–C)** Activity for key enzymes involved in glycolysis pathway. **(D–G)** Expression of protein for key enzymes involved in glycolysis pathway in MT and WT cattle heart. **(H–K)** Expression of phosphorylation of protein for key enzyme protein involved in glycolysis pathway by immunoprecipitation in MT and WT cattle heart. Western blots obtained by immunoblotting antibodies directed against HK2, PFK, PK, and phosphorylation. Abbreviations: PFK, phosphofructokinase; HK2, hexokinase; PK, pyruvate kinase. All data are presented as mean ± SD. Compared with the WT group, *p < 0.05, **p < 0.01, ns: no significant; *t*-tests were used to calculate the *p*-values.

Myostatin plays a decisive role in the regulation of metabolic processes, and the glycolysis in skeletal muscles increases in myostatin-null mouse (Matsakas et al., 2012). In this study, to determine whether glycolysis was affected in cardiomyocytes of cattle with MT, key enzymes of the glycolysis were examined, including HK2, PFK, and PK. Activities of HK2, PFK, and PK were significantly higher in cattle with MT than in WT cattle (Figures 3A–C), although protein expression levels of the three enzymes were not affected (Figures 3D–G). Furthermore, phosphorylation levels of HK2, PFK, and PK were significantly higher in cattle with MT than in WT cattle, with PFK phosphorylation most significantly affected (Figures 3H–K). These results suggested that activity of the glycolysis pathways in cardiomyocytes of cattle with MT increased significantly.

### Myostatin Mutation Activated the PDE5A-cGMP-PKG Pathway

**FIGURE 4:**
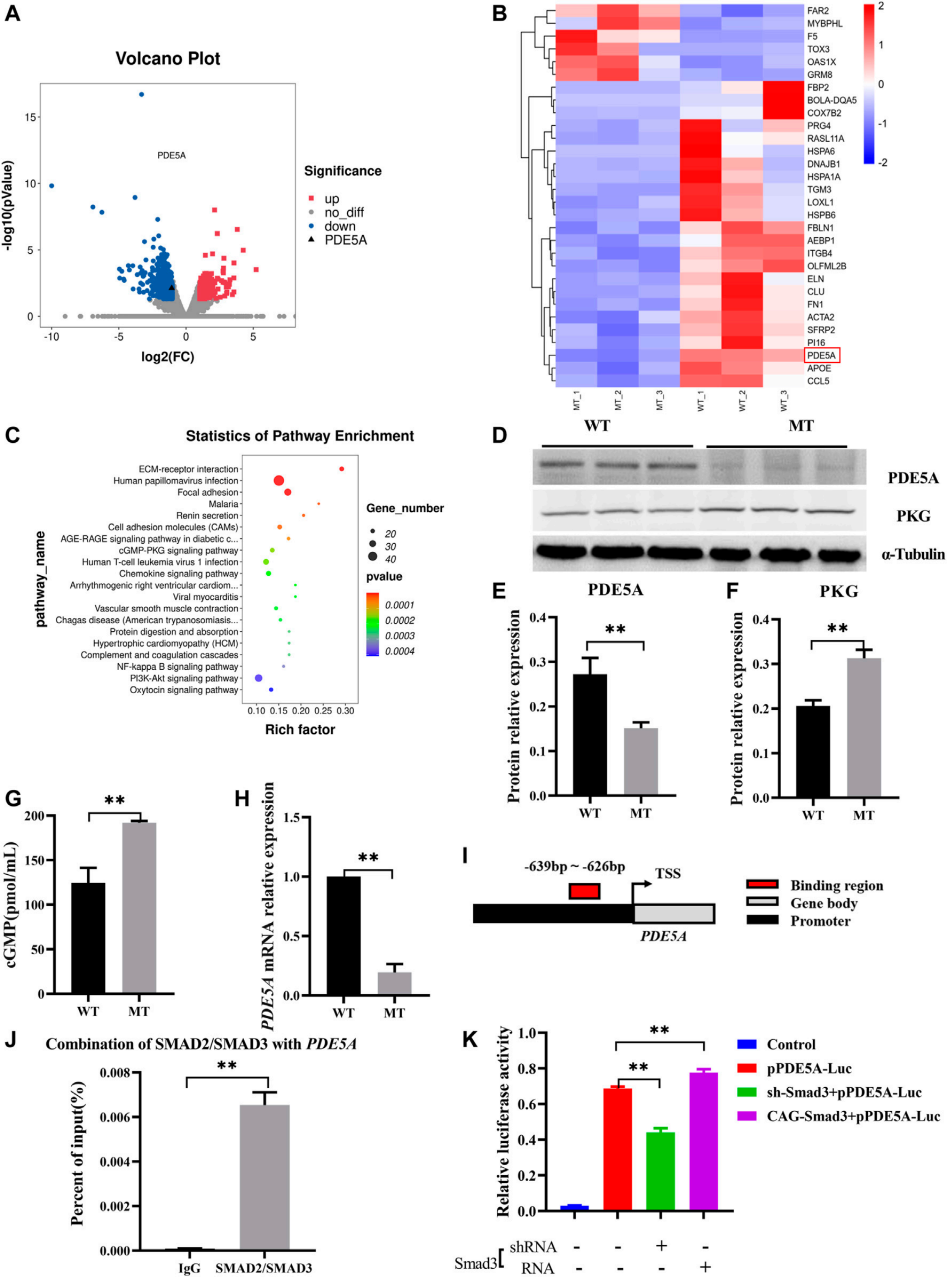
MSTN regulates PDE5A-cGMP-PKG pathway expression *via* SMAD2/SMAD3. **(A)** Volcano plot of differential gene expression analysis. **(B)** Clustering heat map of differential gene expression. **(C)** KEGG analysis of DEGs between WT and MT groups. **(D–F)** Expression of PDE5A and PKG protein in heart of MT and WT cattle. **(G)** cGMP content of MT and WT cattle heart. **(H)** Expression of PDE5A mRNA in heart of MT and WT cattle. **(I)** Schematic representation of SMAD2/SMAD3 binds the *PDE5A* promoter region. **(J)** ChIP-qPCR detected the binding of SMAD2/SMAD3 to the *PDE5A* promoter region in cattle hearts with MT. **(K)** Promoter activity of *PDE5A* was determined by a luciferase assay. All data are presented as mean ± SD. *p < 0.05, **p < 0.01, ns: no significant; *t*-tests were used to calculate the *p*-values.

To further understand changes in expression of the genes in hearts of cattle with MT, transcriptome analysis of WT cattle hearts and those with MT was performed. There were 878 differentially expressed mRNAs [DE mRNAs; *p*-value < 0.05, fold change (FC) > 2] identified between WT hearts and those with MT. Among DE mRNAs, 150 were upregulated and 728 were downregulated (Figures 4A, B and Supplementary Table S3). Analysis of differentially expressed genes (DEGs) revealed that the PDE5A gene was significantly downregulated in cattle with MT (Figures 4A,B). KEGG analysis of DEGs indicated that enrichment occurred in signal pathways such as cGMP-PKG (Figure 4C). Compared with WT cattle, in cattle with MT, PDE5A expression decreased significantly, whereas that of PKG as well as the concentration of cGMP increased significantly (Figures 4D–G). The results indicated that the PDE5A-cGMP-PKG pathway was significantly affected in cattle with MT. Therefore, to determine how MT regulated the PDE5A-cGMP-PKG pathway, expression of mRNA of PDE5A was examined. Expression of *PDE5A* decreased dramatically in cattle with MT (Figure 4H), and the PDE5A-cGMP-PKG pathway was enhanced.

### Myostatin Regulated PDE5A Expression *via* SMAD2/SMAD3

Because MT improved the transcriptional activity of PDE5A, it was hypothesized that MSTN regulates transcriptional activity of PDE5A and that SMAD2 and SMAD3 transcription factors bind with the promoter of PDE5A. Promoter analysis revealed potential binding sites (−639 to −626) in the promoter region of *PDE5A* (NC_037,355.1) (Figure 4I and Supplementary Figure S2A). Chromatin immunoprecipitation coupled with qPCR (ChIP-qPCR) using an anti-SMAD2+SMAD3 antibody revealed that SMAD2/SMAD3 directly bound to the promoter of PDE5A (Figure 4J and Supplementary Figure S2B). To investigate the regulation of PDE5A transcriptional activity by SMAD2/SMAD3, a luciferase assay was performed (Figure 4K). Overexpression of SMAD3 in 293T cells led to increased PDE5A activity, whereas knockdown of SMAD3 decreased expression of PDE5A. These results indicated that SMAD3 activated the transcriptional activity of the PDE5A promoter (Figure 4K).

### Activation of cGMP-PKG Promotes Glycolysis *via* Increasing Phosphorylation of Phosphofructokinase

**FIGURE 5:**
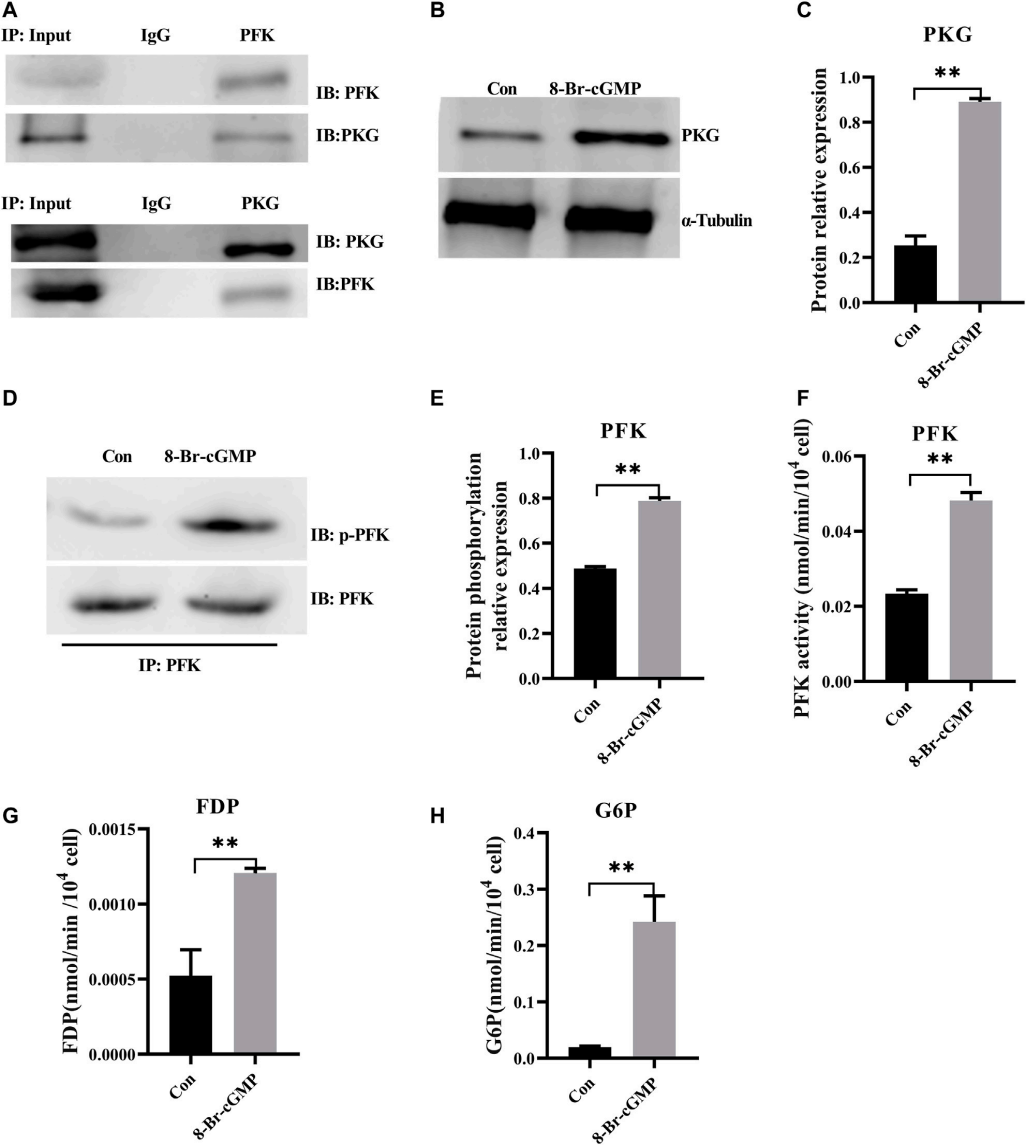
Activation of cGMP-PKG promotes glycolysis *via* increasing phosphorylation of PFK. **(A)** PKG interacts with PFK by co-immunoprecipitation and immunoblotting analyses in cattle heart. **(B,C)** The expression of PKG at the protein level after the activation of 8-Br-cGMP in the H9C2 cells. **(D,E)** Phosphorylation level of PFK protein by immunoprecipitation after the activation of 8-Br-cGMP in the H9C2 cells. **(F)** Activity for PFK enzymes after the activation of 8-Br-cGMP in the H9C2 cells. **(G,H)** Content for key metabolites in glycolysis pathway after the 8-Br-cGMP treatment. FDP, Fructose-1,6 diphosphate; G6P, Glucose-6-phosphate. All data are presented as mean ± SD. Compared with the control group, *p < 0.05, **p < 0.01, ns: no significant; *t*-tests were used to calculate the *p*-values.

As the first characterized effectors for cGMP signaling, PKGs are serine/threonine-specific protein kinases that mediate target activities *via* phosphorylation (Francis et al., 2010). Bioinformatic (GPS 5.0) analysis revealed that there might be potential PKG phosphorylation sites on PFK. Co-immunoprecipitation showed that PKG interacted with PFK (Figure 5A) and that PFK was the target of PKG phosphorylation, which suggested that cGMP-PKG affected key enzymes in glycolysis. In the following experiment, RNA interference was used to stably suppress MSTN expression in H9C2 cells. Glycolytic activities in H9C2 cells were consistent with those in cardiomyocytes in cattle with MT, with glycolysis increasing significantly (Supplementary Figure S3). After treatment of H9C2 cells with PKG activator 8-Br-cGMP, protein expression levels of PKG in treated cells increased significantly (Figures 5B,C), and phosphorylation levels of PFK and its enzyme activity also increased significantly (Figures 5D–F). Metabolites of glycolysis, including fructose-1,6 diphosphate (FDP) and glucose-6-phosphate (G6P), were significantly higher in 8-Br-cGMP-treated cells than in control cells (Figures 5G,H).

### Knockdown of Protein Kinase cGMP-dependent Inhibited Glycolysis *via* Decreasing Phosphorylation of Phosphofructokinase

**FIGURE 6:**
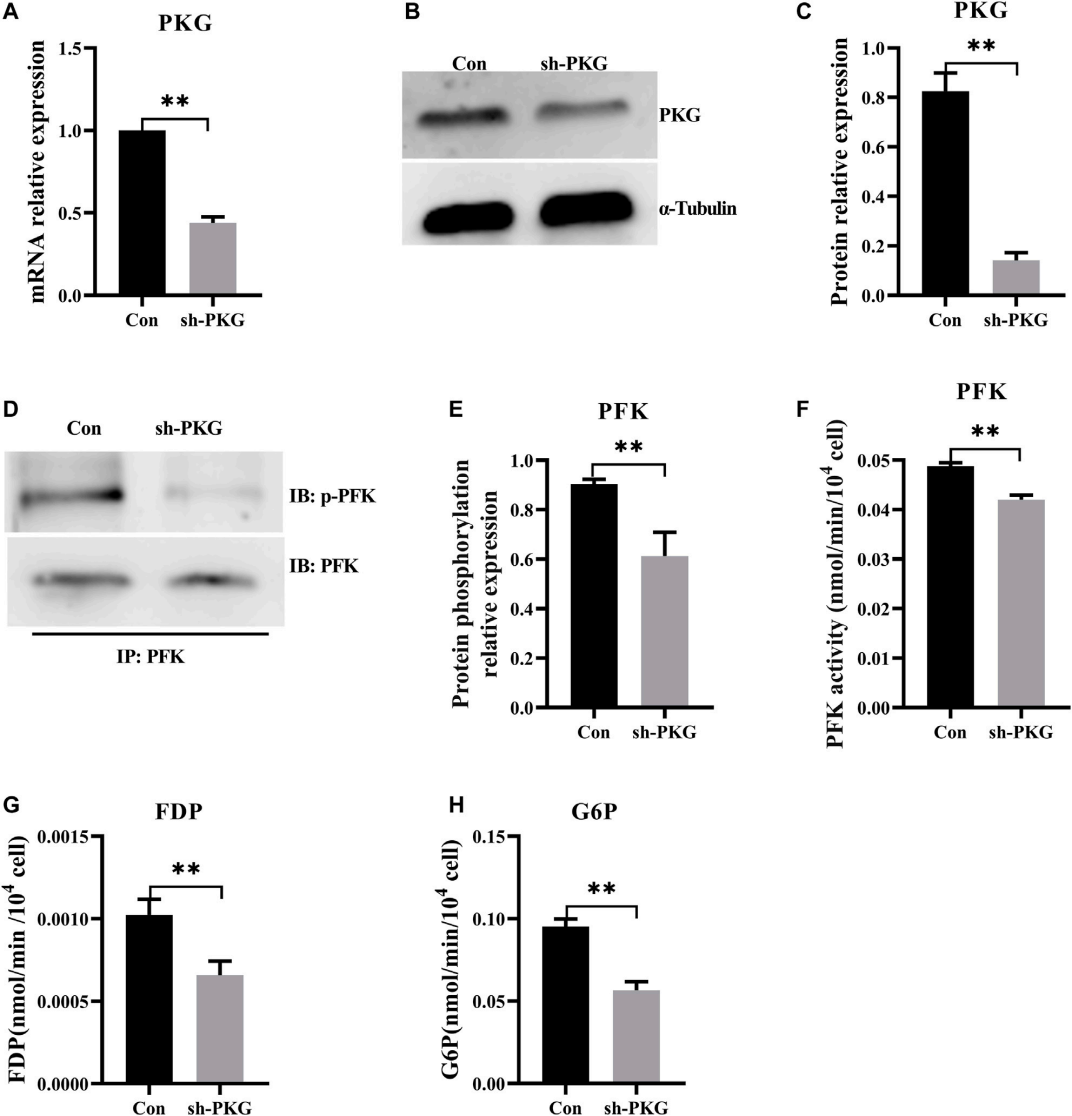
Knockdown of PKG inhibited glycolysis *via* decreasing phosphorylation of PFK. **(A–C)** The expressions of PKG at the mRNA and protein levels after knockdown of *PKG* in the *H9C2* cells. **(D,E)** Phosphorylation level of PFK protein by immunoprecipitation of the *PKG* knockdown *H9C2* cells. **(F)** Enzyme activity of PFK assay in *PKG* knockdown *H9C2* cells. **(G,H)** FDP and G6P, the key metabolites in glycolysis, assayed respectively. Abbreviations: PFK, phosphofructokinase; FDP, Fructose-1,6 diphosphate; G6P, Glucose-6-phosphate. All data are presented as mean ± SD. Compared with the control group, *p < 0.05, **p < 0.01, ns: no significant; *t*-tests were used to calculate the *p*-values.

To knock down PKG in H9C2 cells, highly efficient short hairpin RNA3 was selected from three types of shRNAs. Reverse-transcription qPCR and Western blot (Supplementary Figure S4) respectively confirmed that expressions of PKG at both mRNA and protein levels decreased significantly (Figures 6A–C). Both phosphorylation level and enzyme activity of PFK also decreased significantly in PKG-knockdown cells (Figures 6D–F). In addition, FDP and G6P, the key metabolites in glycolysis, decreased significantly after PKG knockdown (Figures 6G,H).

## Discussion

Myostatin is an effective negative regulator of skeletal muscle growth that is highly conserved in many species from rodents to humans. Myostatin-targeted deletions produce impressive skeletal muscle hypertrophy in beef cattle (Kambadur et al., 1997), humans (Schuelke et al., 2004), sheep (Clop et al., 2006), and dogs (Mosher et al., 2007). Clinically, in certain pathological conditions of the heart (Sharma et al., 1999; Shyu et al., 2006; George et al., 2010), MSTN is also significantly upregulated, indicating that it has a specific role in cardiac pathophysiology. In addition, adult murine hearts exhibit cardiac hypertrophy with genetic inactivation of MSTN (Biesemann et al., 2014), and Jackson et al. (2012) reported that MSTN^−/−^ mice have heavier hearts than the myocardium of WT mice over a wide age range (3–24 months) (Jackson et al., 2012). By contrast, Morissette et al. (2009) found that the cardiomyocytes and hearts of male myostatin-null mice are slightly smaller than those of the littermate controls (Morissette et al., 2009), and in cattle with MT, relative heart size also decreases compared with that of WT cattle (Lekeux et al., 1994). In addition, deficiency of myostatin does not induce ventricular hypertrophy, compared with wild-type mice (Cohn et al., 2007; Heineke et al., 2010; Butcher et al., 2017). In the present study, MT had no effect on bovine cardiac hypertrophy, and no obvious exterior morphological abnormalities were observed. The ratio of HW/TL in MT cattle was similar to that of WT cattle, whereas the ratio of HW/BW of WT cattle was greater than that in cattle with MT. Myostatin mutation increased skeletal muscle weight, leading to a denominator that was larger when normalizing HW to BW, necessitating the use of tibia normalization. Heart weight and volume and ventricular wall thickness, atrium wall thickness, and interventricular septum and interatrial septum wall thickness of cattle with MT were greater than those of WT cattle. Myostatin mutation did not change the composition of cardiac collagen, as reported previously (Cohn et al., 2007). These results implied that the cardiac phenotype and cardiomyocytes might be under a different Myostatin mutation environment. Moreover, we demonstrated that Myostatin mutation promotes myoblast differentiation through SMAD2/3 in cattle, leading to skeletal muscle hypertrophy (Gao et al., 2020). Choi et al. (2019) reported that inhibition of MSTN induces skeletal muscle hypertrophy through upregulation of Akt/mTOR pathway *via* Smad (Choi et al., 2019). These regulators crosstalk with each other in heart development and need to be further studied.

Myostatin is involved in the regulation of energy metabolism, and deficiency in myostatin function increases glucose utilization and improves insulin sensitivity (Gonzalez-Cadavid and Bhasin, 2004; Guo et al., 2009; Wilkes et al., 2009). Liu et al. (2018) reported that MSTN disrupts insulin-receptor downstream signaling pathways through IRS-1/PI3K/Akt, insulin-induced activation of AMPK, and translocation of Glut4 (Liu et al., 2018). Furthermore, MSTN decreases insulin sensitivity by inducing degradation of insulin receptor substrate-1 in C2C12 cells (Bonala et al., 2014). In addition to reduction in overall body fat mass, MSTN inactivation reduces intramuscular fat content in meat to some extent (Kärst et al., 2013; Qian et al., 2015). In addition, in transgenic mice overexpressing the inhibitory propeptide domain of MSTN, adipose content and resistance to diet-induced obesity decrease significantly (Zhao et al., 2005). Myostatin may also affect metabolism of adipose tissue by inhibiting differentiation of pre-adipocytes, and thus, both lipid accumulation and myostatin deficiency may promote browning of adipocytes (Li et al., 2011; Braga et al., 2013; Zhu et al., 2015). Ren et al. (2020) identified a novel MSTN signal that regulates fat deposition by affecting fatty acid desaturation in MSTN-Knockout pigs *via* MEF2C/miR222/SCD5 (Ren et al., 2020). Myostatin also has a role in regulation of myocardial metabolism. Conditional inactivation of MSTN in adult murine hearts leads to increases in glycolysis and glycogen storage (Biesemann et al., 2014). In the present work, MT significantly increased levels of metabolites and activities of key enzymes involved in glycolysis in the bovine heart. These results suggest that the MSTN signaling pathway plays a crucial role in regulating glycolysis in the heart.

Zhang and Kass (2011) confirmed that the activation of the cGMP/PKG signaling pathway contributes to the adaptation and protection of myocardial postconditioning to a certain extent, alleviates cardiac stress responses, attenuates pathological hypertrophy, protects against ischemic injury, and increases cell survival (Zhang and Kass, 2011). The cGMP-hydrolyzing phosphodiesterase enzymes (PDEs) can decompose cGMP and therefore are the primary regulators of intracellular cGMP level (Francis et al., 2011). Members of the PDE family catalyze the hydrolysis of 3′,5′-cyclic nucleotides to the corresponding inactive nucleoside 5′-monophosphates (Francis et al., 2011). Although there are seven isoforms of PDEs in myocytes (including PDE1, 2, 3, 4, 5, 8, and 9), PDE5 is specific to cardiomyocytes, was discovered first, and remains the best characterized (Kim and Kass, 2017). Activating PDE5A inhibits levels of both cGMP and PKG; whereas inhibiting PDE5A causes the opposite effect (Ranek et al., 2019). Furthermore, cGMP converts inactive PKG to activated PKG (Lee and Kass, 2012). The enzyme PKG is a serine/threonine-specific protein kinase tnat phosphorylates serine and threonine residues on numerous cytosolic proteins, including cardiac myosin-binding protein C (Thoonen et al., 2015), transient receptor protein channels 3 and 6 (Koitabashi et al., 2010), L-type calcium channel (Schröder et al., 2003), Na^+^/H^+^ exchanger (Kilic et al., 2005), p38 (Browning et al., 2000), Orai1 (Wang et al., 2015), and β-catenin (Thompson et al., 2000). Moreover, PKG regulates the activity of many transcription factors in different types of cells, including cAMP-response element binding protein, GATA4, transcription factor II-I, and nuclear factor-kappa B (Gudi et al., 2000; Casteel et al., 2002; He and Weber, 2003; Pilz and Casteel, 2003; Ma et al., 2016). Therefore, PKG is involved in many cellular functions, including differentiation, apoptosis, relaxation, remodeling, hypertrophy, neuronal plasticity, platelet aggregation, protein degradation, and erectile dysfunction (Hedlund et al., 2000; Persson et al., 2000; Schlossmann and Desch, 2011; VerPlank et al., 2020). Specifically, in the cardiovascular system, PKG not only regulates vascular endothelial cell motility, migration, and proliferation but also negatively modulates cardiac myocytes contractility and hypertrophy, as well as mediating apoptosis (Schlossmann and Desch, 2011). However, there are few reports on involvement of cGMP/PKG pathways in metabolism, although Tian et al. (2018) reported that the cGMP/PKG pathway regulates stimulation of glucose transport and uptake by NO (Tian et al., 2018). In this study, MT inhibited PDE5A transcription by SMAD2/SMAD3 and increased levels of cGMP and expression of PKG. Myostatin mutation also promoted glycolysis pathways. These results showed that MSTN might play a regulatory role in glycolysis in the heart *via* the PDE5A-cGMP-PKG pathway. Furthermore, results suggested that PKG upregulated the phosphorylation level of PFK. Thus, MT may potentiate PFK phosphorylation *via* the PDE5A-cGMP-PKG pathway and thereby promoted glycolysis in the bovine heart.

To further evaluate the role of PKG during glycolysis, the activator 8-Br-cGMP was used to activate PKG in H9C2 cells. Phosphorylation of PFK increased significantly and levels of glycolytic metabolites increased, indicating promotion of glycolysis. However, with knockdown of PKG in H9C2 cells, phosphorylation of PFK decreased, and levels of glycolysis metabolites decreased significantly, indicating inhibition of glycolysis. These results further demonstrated that MT promoted glycolysis by increasing phosphorylation level of PFK, *via* the upregulation of PKG. Previously, Biesemann et al. (2014) found that MT leads to an increase in glycolysis *via* the TAK1-AMPK pathway in the murine heart (Biesemann et al., 2014). Therefore, interaction between the TAK1-AMPK pathway and the PDE5A-cGMP-PKG pathway might be a very important role in MSTN-induced glycolysis in the heart. However, this supposition requires further investigation.

**FIGURE 7:**
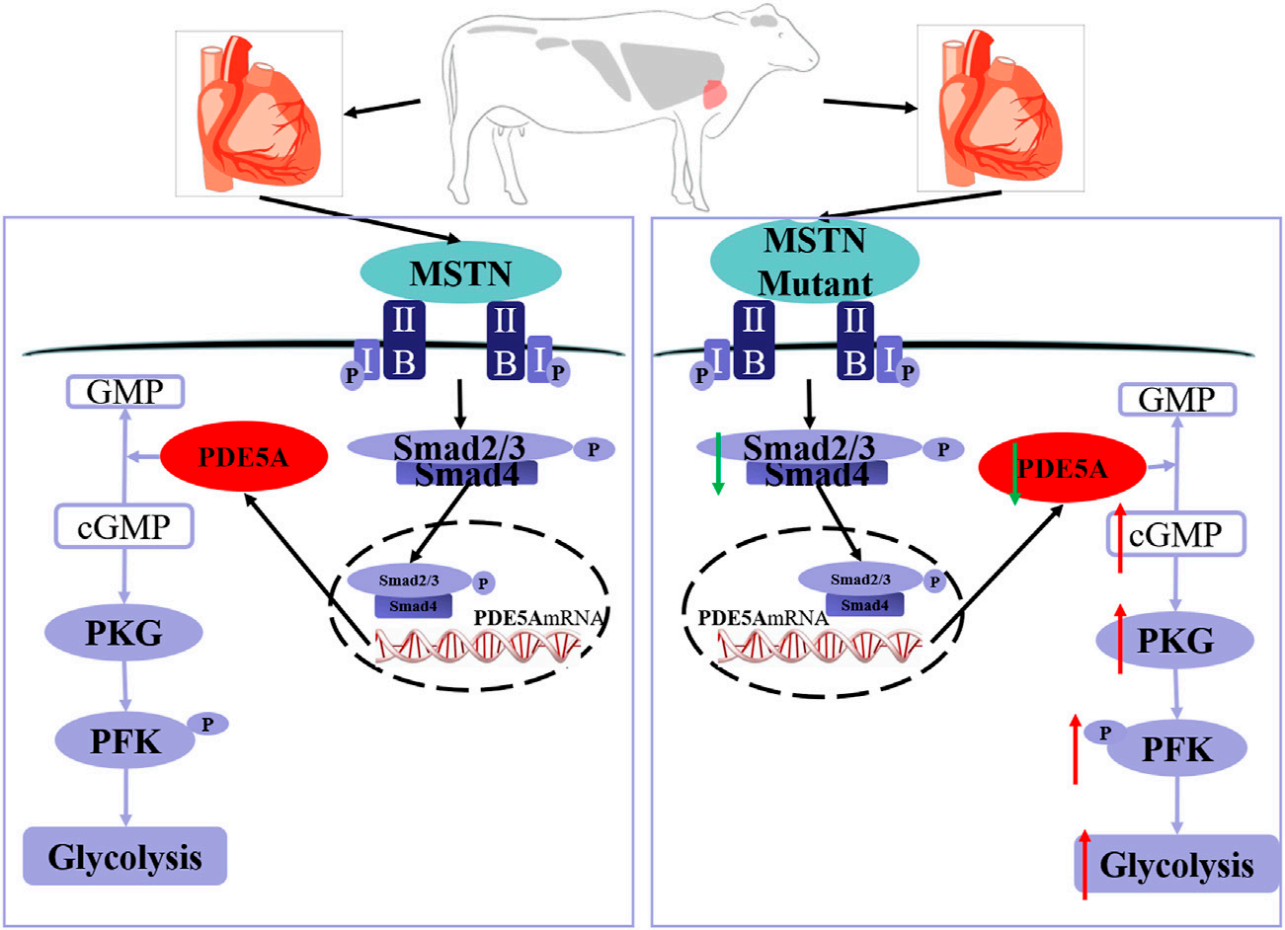
Schematic of mechanisms for the activation of PDE5A-cGMP-PKG signaling and the promotion of glycolysis by MSTN. MSTN activate the pathway *via* combining with ActRIIB, which then binds to the type I receptor to form a receptor complex. After the receptor complex is activated by the signal, SMAD2/SMAD3 is activated *via* phosphorylation. Then, SMAD2/SMAD3 will detach from receptor complex and bind to SMAD4. Then, the heteromeric complex enters the nucleus, binds to the promoter region, and alters transcription of PDE5A gene. The left one is the wild-type cattle and the right one is the MSTN mutant cattle. The MSTN mutant decreased SMAD2/3 expression, which led to transcription downregulation of PDE5A and thereby promoted the cGMP/PKG pathway, increased the phosphorylation level of PFK, and subsequently enhanced glycolysis. The red arrow indicates upregulation and the green arrow indicates downregulation.

In conclusion, MT increases the phosphorylation level of PFK, which leads to an increase in glycolysis in the bovine heart. As indicated by the model in Figure 7, MT decreases expression of SMAD2/3, which binds directly to the PDE5A promoter region that in turn inhibits PDE5A transcription. Then, the cGMP/PKG pathway is activated, and the phosphorylation level of PFK increases, leading to a subsequent increase in glycolysis.

## Data Availability

The datasets presented in this study can be found in online repositories. The names of the repository/repositories and accession number(s) can be found below: https://bigd.big.ac.cn/gsa/browse/CRA004316, CRA004316.

## Abbreviations

MSTN, myostatin; MT, MSTN gene mutant; WT, wild type; PDE5A, phosphodiesterase 5A; cGMP, 3′,5′-cyclic guanosine monophosphate; PKG, protein kinase cGMP-dependent; MYH7, myosin heavy chain 7; ANP, natriuretic peptide A; PFK, phosphofructokinase; HK2, hexokinase2; PK, pyruvate kinase; FDP, Fructose-1,6 diphosphate; G6P, Glucose-6-phosphate.
