# Supplementary material for: Myostatin Mutation Promotes Glycolysis by Increasing Phosphorylation of Phosphofructokinase via Activation of PDE5A-cGMP-PKG in Cattle Heart
Source: Front Cell Dev Biol. 2022 Jan 28;9:774185. doi: 10.3389/fcell.2021.774185 (PMC8831326; doi:10.3389/fcell.2021.774185)
Supplement: Supplementary file 4 [file DataSheet1.docx]

Supplementary Material

## Supplementary Figure


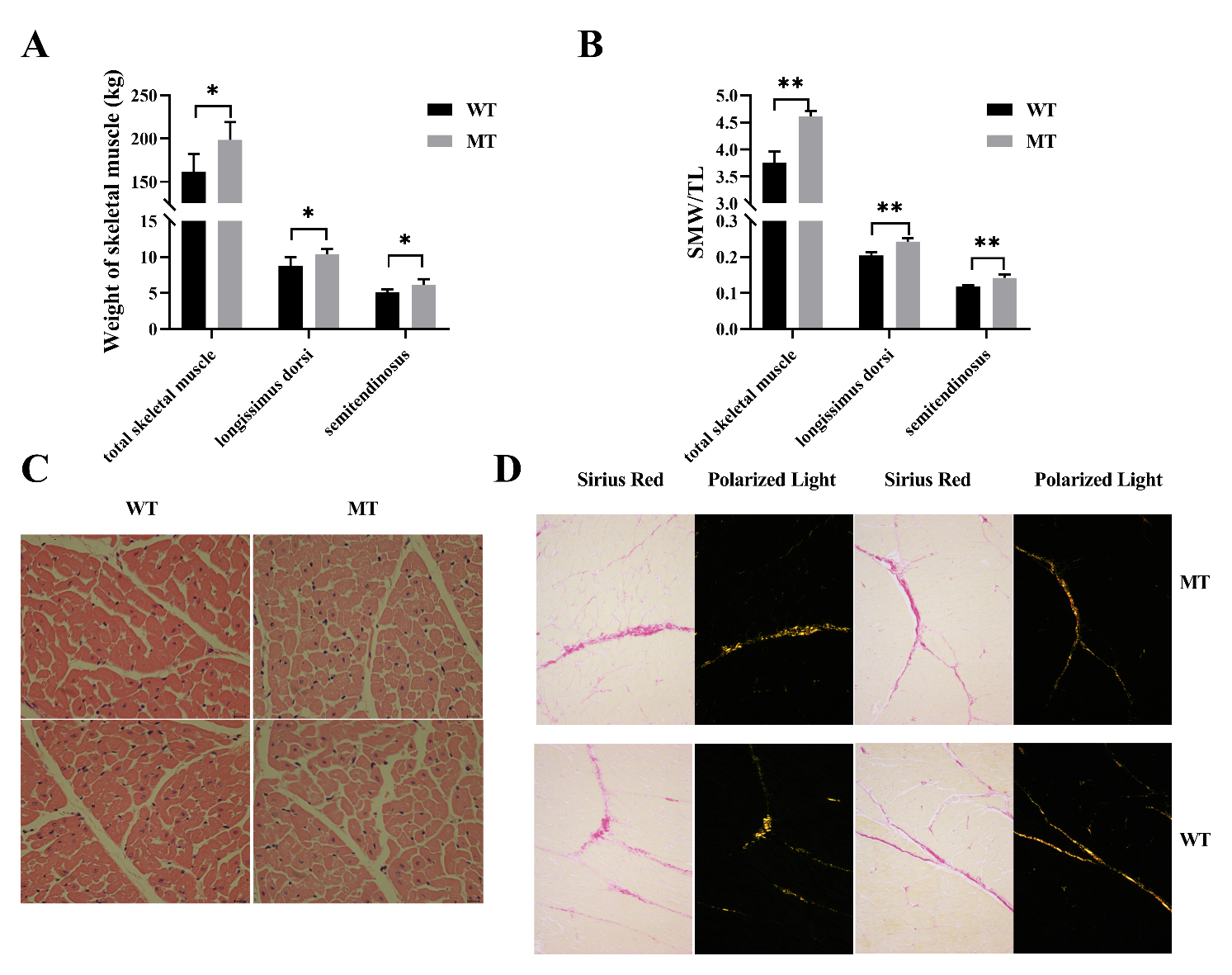


**Supplementary Figure 1.** Weight of skeletal muscle, hematoxylin and eosin (H&E) staining of heart and sirius red stained of heart from the MT and WT cattle. **(A,B)** Total skeletal muscle, longissimus dorsi and semitendinosus weight in WT and MT cattle. **(C)** HE staining of heart samples (400×). **(D)** Sirius red stained sections and polarized light microscopy from MT and WT cattle heart. WT: wild type; MT: Myostatin mutation.





**Supplementary Figure 2.** SMAD2/SMAD3 combined with the promoter of *PDE5A*. **(A)** The motif of SMAD2/SMAD3 and the predicted results for the combination of SMAD2/SMAD3 with the promoter of *PDE5A*. **(B)** Amplification of *PDE5A* by SMAD2/SMAD3 combination.





**Supplementary Figure 3.** Knockdown of MSTN increased glycolytic capacity of H9C2 cells. **(A)** Expression of MSTN mRNA after knockdown of MSTN in H9C2 cells. **(B)** Expression of MSTN protein after knockdown of MSTN in H9C2 cells. **(C)** Representative expression of MSTN immunofluorescence images after knockdown of MSTN in H9C2 cells (green, MSTN; blue, nuclei; scale bar, 100 μm). **(D)** Activity for PFK enzymes after knockdown of MSTN in the H9C2 cells. **(E, F)** Content for key metabolites in glycolysis pathway after knockdown of MSTN in the H9C2 cells. All data are presented as mean ± SD. Compared with the control group, **P*<0.05, ***P*<0.01, ns: no significant; *t*-tests were used to calculate the *p*-values. Abbreviations: PFK, phosphofructokinase; FDP, Fructose-1,6 diphosphate; G6P, Glucose-6-phosphate.


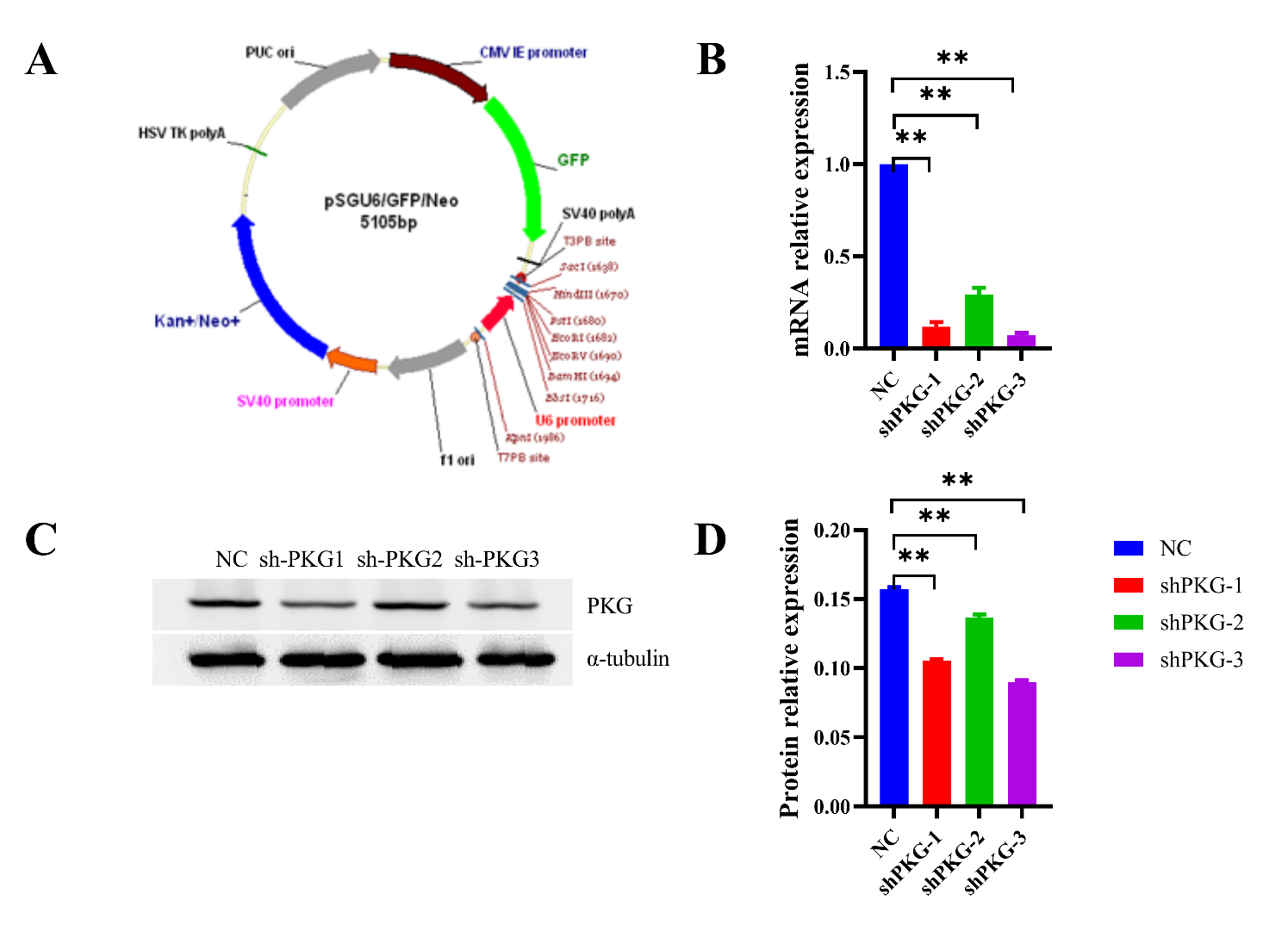


**Supplementary Figure 4. Selection of high-efficient shRNA. (A)** Vectors used for knockdown of PKG. **(B)** Expressions of PKG mRNA from three types of shRNAs. **(C, D)** Expressions of PKG proteins from three types of shRNAs. All data are presented as mean ± SD. Compared with the NC group, **P*<0.05, ***P*<0.01, ns: no significant; *t*-tests were used to calculate the *p*-values.
